# Prevalence, Correlates, and Barriers of Contraceptive Use among Women Attending Primary Health Centers in Aljouf Region, Saudi Arabia

**DOI:** 10.3390/ijerph17103552

**Published:** 2020-05-19

**Authors:** Doaa M. Abdel-Salam, Ibrahim A. Albahlol, Reem B. Almusayyab, Nouf F. Alruwaili, Manar Y. Aljared, Maram S. Alruwaili, Renad M. Alnasser

**Affiliations:** 1 Family and Community Medicine Department, College of Medicine, Jouf University, Aljouf 42421, Saudi Arabia; 2 Public Health and Community Medicine Department, Faculty of Medicine, Assiut University, Assiut 71526, Egypt; 3 Obstetrics and Gynecology Department, College of Medicine, Jouf University, Aljouf 42421, Saudi Arabia; iaelbahloul@ju.edu.sa; 4 Obstetrics and Gynecology Department, College of Medicine, Mansoura University, Dakahlia Governorate 35516, Egypt; 5 College of Medicine, Jouf University, Aljouf 42421, Saudi Arabia; Reemalmusib778@gmail.com (R.B.A.); nouf.alr18@gmail.com (N.F.A.); manar1419m@gmail.com (M.Y.A.); maramalruwaili6@gmail.com (M.S.A.); renad.1998r@gmail.com (R.M.A.)

**Keywords:** contraceptive use, prevalence, correlates, barriers, Saudi women

## Abstract

(1) *Backgrounds and Objectives*: with the rapid alteration in the socio-demographic pattern of the Saudi community, particularly the changes concerned with women’s education and work force, contraceptive use must be a fundamental aspect in the life of women in reproductive age. The present study aimed to identify the prevalence and correlates of contraceptive use among women attending primary health centers in Aljouf region, Saudi Arabia, and to determine the perceived barriers of stopping or not using contraceptive methods in this population. (2) *Methods*: a primary health center-based cross-sectional study was conducted among 369 women of reproductive age. Data collection was done by using structured questionnaires distributed during face to face interviews with the participants. Data were analyzed using the SPSS program, version 24. (3) *Results*: most of the participants (n = 166; 45%) were current contraceptive users and 32.2% (n = 119) reported previous use of contraceptive methods. Pills were the most frequently used method (n = 203; 71.2%) and intrauterine devices (IUDs) came next (n = 67; 23.5%) while surgery was the least common method among the respondents (n = 3; 1.1%). Logistic regression analysis showed that the significant predictors of contraceptive use were: age > 35 years (odds ratio (OR): 4.52; confidence interval (CI): 1.56–15.42), Children number ≥ 4 (odds ratio (OR): 1.41; confidence interval (CI): 1.06–1.92) and monthly income ≥ 5000 Saudi Riyal (RS) (odds ratio (OR): 2.29; confidence interval (CI): 1.24–8.27). The most perceived barriers towards contraceptive utilization were cultural, demographic, medical, administrative, and barriers related to the method itself. The least reported barriers were psychosocial and physical. (4) *Conclusions*: the present study showed a high prevalence of contraceptive use among Saudi women in Aljouf region, Saudi Arabia. The study recommended sustained efforts to increase population awareness of the importance of family planning. Policymakers should discover the barriers that prevent contraceptive utilization by women.

## 1. Introduction

Contraceptive use permits women in reproductive age to reach their desired number of children and plan the intervals between pregnancies [1]. The prevalence of contraceptive use is defined as “the percent of women of reproductive age who are utilizing (or whose sexual partner is utilizing) a contraceptive method at any particular point of time, almost always calculated for married women” [2]. The marked increase in contraceptive use in developing countries in the past few years has been correlated with a reduced percentage of unintended pregnancies and the reduction of maternal mortality by 40% [3]. In addition to the benefits to women, regulation of inter-pregnancy intervals will lead to better perinatal outcomes [3].

The Middle East has shown rapid changes in the socio-demographic features of its population. Women are the most encountered in these changes as most of them had attained high education and joined the workforce. So, there were changing behaviors and attitudes towards fertility with more couples adopting family planning [4].

According to Saudi Household Health Survey, the prevalence of contraceptive use was 30.4% in 2018 [5]. In Saudi Arabia, contraceptive use was influenced by numerous factors such as working conditions, maternal age, educational level, parity, family size, and gender of the last child [6,7,8]. The most common contraceptive methods used in Saudi Arabia were oral and intrauterine contraceptives [9,10,11]. With the current Saudi vision of 2030, contraception is considered to have a fundamental role in reproductive health improvement and women’s empowerment [12]. Family planning is not extensively used in Saudi Arabia [5] and the prevalence of contraceptives use is low compared with other developing countries [13,14]. The decision to use or not to use contraceptive methods is dependent mainly on numerous barriers that arise from administrative, cultural, cognitive, and psychosocial factors as well as physical barriers and barriers related to the method itself [15]. Program managers and policymakers can strengthen family planning programs by working to reduce the barriers that prevent women from choosing and using different contraceptive methods [16].

In Saudi Arabia, studies on contraceptive use are deficient and no study has been conducted on barriers towards contraceptive utilization. To the best of authors’ knowledge, no study has been carried out in Aljouf region located in the northern part of Saudi Arabia regarding contraceptive use. So, the present study was done aiming to identify the prevalence, correlates, and barriers of contraceptive use among Saudi women attending primary health centers in Aljouf region, Saudi Arabia. This information may be beneficial to health care managers to plan effective strategies for the promotion of contraceptive use in Saudi Arabia.

## 2. Methodology

### 2.1. Study Design and Setting

A cross-sectional study was carried out to identify the prevalence, correlates and barriers of contraceptives use among women attending primary health centers in Aljouf region, Saudi Arabia. Aljouf region is located in the northern part of Saudi Arabia. According to the estimated census of 2018, Aljouf region has a total population of 520,737. Aljouf region has four governorates: Skaka, Domat Al-Jandal, Alqurayat, and Tabargel. The present study was conducted in the primary health centers of Aljouf region. The participants were selected from the waiting areas in the primary health centers and briefed on the aim and objectives of the study. The researchers were always available to respond to participants’ inquiries and comments. Data collection was done from July to December 2019.

### 2.2. Sample Size

Sample size estimation was done using n = P (1−P) z^2^/d^2^ assuming the prevalence of contraceptives use to be 68% [17], Z = 1.96 and d = 0.05, applying a confidence level of 95% and 80% power of the study. The estimated sample size was 335. After adding 10% as a non-response rate, the sample size was raised to 369.

### 2.3. Sampling Technique

There were 43 primary health centers in Aljouf region, Saudi Arabia. A simple random sampling technique was done to pick 10 primary health centers out of 43. The number of women selected in each primary health center was proportional to the number of women served by this center until reaching the desired sample size.

### 2.4. Inclusion Criteria

All married Saudi women aged 18–49-year-old who had not reached menopause attending the health centers were invited to participate in the present study and fill the questionnaire during face to face interviews with them.

### 2.5. Exclusion Criteria

Women who did not fulfill the above criteria were excluded from the present study.

### 2.6. Data Collection Tool

A structured interview questionnaire was used by the researchers during face to face interviews with the participants. The questionnaire was validated in two previous studies [13,18]. The questionnaire was composed of three parts. The first part inquired about participants’ socio-demographic information such as age, residence, education, occupation, marriage duration, children number, husband’s age, and monthly income. The second part was about the utilization of contraceptive methods (current/previous use of contraceptive methods), types of contraceptive methods used, and sources of information about different contraceptive methods. Current use of the contraceptive method can be defined as the use of any modern (oral contraceptive pills, intrauterine device (IUD), male condom, injection, and any surgical procedures) or traditional (the withdrawal and rhythm method) method to avoid or delay pregnancy within the previous 30 days while previous use denotes having any contraceptive method more than one month beforehand [19]. The third part was about barriers to stop or not use contraceptive methods. Women could choose any number of barriers. Their response was recorded as agreeing or disagreeing with a certain barrier. These barriers could be classified as follows:

- Cultural barriers (thinking that contraceptive utilization is bad behavior/childbearing is an easy process at a young age/nontraditional method can harm women health/absence of female physician/ideal number of children is not known),
- Demographic barriers (desire to have more children because of low parity/contraceptive method should be used by old women/difficulty in getting pregnant),
- Psychosocial and physical barriers (someone prevents you from using contraception/involvement in many activities throughout the day/primary health center is away from the home),
- Medical barriers (requesting the women to return more for examination/informed consent from the husband is mandatory/hormonal contraception requires the women to be on her period),
- Administrative barriers (did not see any announcement or attend an educational session about the family planning/poor quality service/negative attitude of health care provider/previous bad experience/no privacy during examination), and.
- Contraceptive method barriers (desire to have a more efficient contraceptive method/Failing in using different contraceptive methods/Encountered serious side effects).

A pilot study was conducted on 30 women to test the clarity of the questionnaire. No modifications were done on the used questionnaire. Results of the pilot study were not included in the present study.

### 2.7. Data Analysis

SPSS program, version 24 (SPSS Inc., Chicago, IL, USA) was used for data analysis. Descriptive statistics were performed using number and percentage for categorical variables, mean ±SD for continuous variables. Factors associated with contraceptive use were identified using Chi-square and Fisher’s Exact tests. Logistic regression analysis was done to adjust for confounding variables. *p*-value < 0.05 was considered to be statistically significant.

### 2.8. Ethical Considerations

The proposal of this study was submitted to the Ethical Review Committee of Jouf University, Saudi Arabia, and data collection was commenced after ethical clearance (Approval No: 2-19-6/40). Informed written consent was taken from all women who agreed to participate in the current study. The questionnaires were distributed anonymously to ensure the privacy and confidentiality of the collected data.

## 3. Results

**Figure 1:**
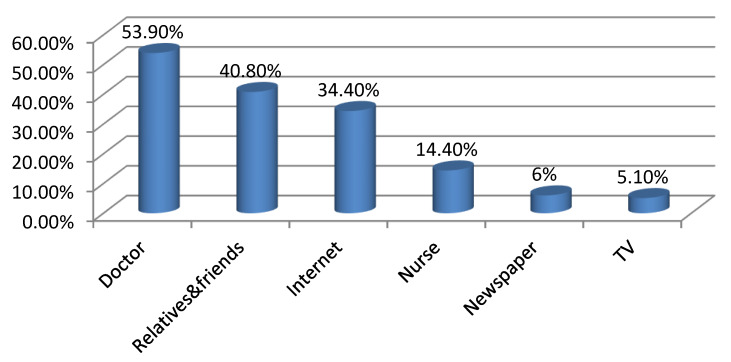
Sources of contraceptive information among women attending primary health centers in Aljouf region, Saudi Arabia. *Notes*: More than one answer had been reported.

**Figure 2:**
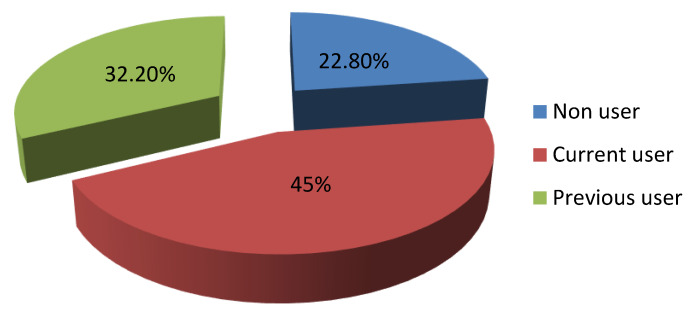
Prevalence of contraceptive use among women attending primary health centers in Aljouf region, Saudi Arabia.

**Figure 3:**
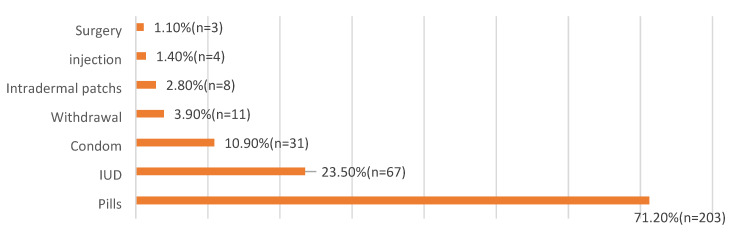
Distribution of different contraceptive methods among women attending primary health centers in Aljouf region, Saudi Arabia. *Notes*: More than one answer had been reported.

Table 1 describes the socio-demographic features of the participants. A total of 369 women attending primary health care centers were recruited in this study. Their mean age was 34.09 ± 6.42 years. The majority of the respondents were urban residents (96.2%), employed (52.6%), university graduates or higher (75.3%) and had from 1 to 3 children (51.8%). The duration of marriage was more than 15 years among 41.7% of the participants. Monthly income was 5000 RS or more among 84.3% of the respondents. On inquiry about the source of contraceptive knowledge among the respondents, the main source was attending doctors (53.9%) followed by relatives and friends (40.8%) while only 5.1% had their orientation from TV (Figure 1). Figure 2 showed that 45% (n = 166) of the participants were currently utilizing a contraceptive method while 32.2% (n = 119) experienced a history of contraceptive utilization. Only 22.8% (n = 84) of the participants did not use contraceptive methods. Oral contraceptive pills were the dominant utilized method for family planning (71.2%) and intrauterine devices (IUDs) came next (23.5%) while surgery was the least method among the respondents (1.1%) (Figure 3).

Table 2 investigates the correlates of contraceptive use among the respondents. There was a statistically significant association between contraceptive use and women age, children number, years of marriage, and monthly income. Logistic regression analysis showed that women’s age > 35 years, children number ≥ 4, and monthly income ≥ 5000 RS were significant predictors of contraceptive use. Women aged > 35 years were 4.5 times more likely to use contraceptive methods than women aged < 25 years (odds ratio (OR): 4.52; confidence interval (CI): 1.56–15.42). Furthermore, women with a number of children ≥ 4 were 1.4 times more likely to use contraceptive methods than women with no children (odds ratio (OR): 1.41; confidence interval (CI): 1.06–1.92). The present study also revealed that women with monthly income ≥ 5000 RS were 2.3 times more likely to use contraceptive methods than women with monthly income < 5000 RS (odds ratio (OR): 2.29; confidence interval (CI): 1.24–8.27).

The perceived barriers towards contraceptive use among non-users and previous users were illustrated in the Table 3. Cultural barriers were prevalent as 98% thought that nontraditional contraceptive methods can harm women’s health and 79.8% believed that childbearing is an easy process at a young age. Regarding medical barriers, 75.4% reported that informed written consent from the husband is mandatory to have contraceptive methods while 64.5% believed that hormonal contraception requires the women to be on her period. Low parity and desire to have more children were reported by 61.1%. As regard barriers related to the method itself, 47.3% claimed the development of serious side effects while 45.8% said they seek to have more efficient contraceptive methods. Concerning administrative barriers, 63.1% did not attend any educational session about the use of contraception and 38.4% thought of bad quality of service. No time available because of continuous involvement throughout the day was reported by 40.9% while 28.1% claimed that they were obliged to the non-utilization of a family planning method.

## 4. Discussion

Contraceptive methods have gained more and more interest throughout the world as they protect both mothers and children against different morbidities and mortalities related to repeated pregnancies together with other socioeconomic advantages [19,20]. The issue of family planning is a thorny subject in the Middle East with special concern to Gulf areas owing to cultural and religious aspects [21]. There is a high incidence of polygamy in the Saudi community which is legalized. Men feel proud of having more and more children in this community. This encourages wives to have more children to maintain their marital relationship which represents a barrier against contraceptive use in this population [22]. Hence, knocking out the issue of contraceptive use is of the utmost value in favor of improving the community health standards and adding to the existing literature. Therefore, the present study was conducted to identify the prevalence, correlates and barriers toward contraceptive use among the targeted population. This study demonstrated that 45% of the respondents were currently using a contraceptive method while 22.8% were not. The socioeconomic pattern of the Saudi community was changed rapidly in the last years because of women’s education and employment with accompanying changes in fertility beliefs and behaviors and consequently more tendencies to birth spacing with different contraceptive methods. These figures nearly came in correspondence with other studies that were reported by Alsheeha who studied the issue at Al-Qassim area, Saudi Arabia and Arbab et al. who carried out their study among Qatari women [7,23]. However, lower contraceptive prevalence rate was observed among Yemeni women (27.7%) [24]. A higher rate of contraceptive use was reported by Al Kindi and Al Sumri who showed that 54% of Omani women were currently utilizing a family planning method and 76.8% experienced previous usage of a contraceptive method [13]. In addition, the prevalence of previous and current contraceptive use were 68.0% and 61.8%, respectively among mothers of reproductive age in Ajman, United Arab Emirates [14]. Much higher contraceptive use was observed in Eastern Asia (82%), Northern Europe (77%), and North America (75%) [24]. This marked variation of contraceptive utilization could resort to a lot of factors mainly traditional concepts, cultural aspects, and sometimes religious concerns. The present study clarified the role of doctors as the common source of awareness of contraceptive methods followed by friends and relatives while TV and newspapers played a minor role. This confirmed the pivot role of the doctor in rising and improving community awareness as they are well trained, supply right and suitable counseling and correct misbelieves regarding contraceptive methods. In agreement with the present findings, other studies reported that doctors were the main source of contraceptive knowledge [13,14,21]. Friends and relatives were reported to be the most common source of contraceptive orientation by other studies [7,23]. In the present study, the most preferred contraceptive method was shown to be oral contraceptive pills (71.2%) followed by IUDs (23.5%) and condom (10.9%). This is in line with other Saudi studies that have revealed the popularity of oral contraceptive pills [7,25,26]. The high prevalence of modern contraceptive methods use among participants in this study was also observed in other studies [14,23], probably because of the effect of economic development and increased availability of information. However, a high prevalence of traditional contraceptive methods use was shown in other studies [13,21]. Women’s age, duration of marriage and parity were significant predictors of contraceptive use in this study. Contraceptive use was more common among women older than 40 years, married for more than 15 years, and with higher parity, maybe because these women have already achieved their fertility goals and reached the desired number of children. These findings were consistent with other studies [7,13,14,23]. In addition, contraceptive use was significantly higher among women with a high monthly income in agreement with other studies [7,13]. High monthly income affects economic development and leads to the easy availability of information and consequently easy accessibility to these contraceptive methods.

The present study revealed different barriers to contraceptive use among non-users and previous users. Prevalent barriers towards contraceptive use in this study were cultural, demographic, medical, administrative, and barriers related to the method itself. The least reported barriers were psychosocial and physical. These findings were in agreement with that reported by Eltomy et al. who investigated barriers affecting the utilization of family planning services among Egyptian women [18]. Studies conducted in Nepal and Jordan showed that the administrative barriers were prevalent because of problems in the health care delivery system and lack of proper counseling by health care providers [27,28]. However, findings of the present study were inconsistent with study conducted in Pakistan which showed that psychosocial barriers were the dominant barriers to contraceptive utilization [29]. The variation of reported barriers towards contraceptive use among different studies could be attributed to cultural and personal characteristics as well as subjective perceptions. Regarding administrative barriers in the present study, two-thirds did not attend any educational sessions about family planning. These administrative barriers indicate a lack of awareness about family planning which may be a reason for not using contraceptive methods among participants of the present study. This was shown in a study conducted in Sub-Saharan Africa which revealed that lack of awareness of family planning was the main reason for the non-utilization of contraceptive methods [30]. Concerning cultural barriers in this study, nearly all the participants thought that nontraditional contraceptive methods could harm women’s health. This is because of a lack of awareness of participants about the medical benefits of some contraceptive methods such as diminution of ovarian cancer with the use of combined oral contraceptive pills and treatment of abnormal uterine bleeding with the use of hormone loaded IUD. However, other studies indicated that the absence of a female physician was the most reported cultural barrier among the participants [18,28]. Desire to have more children because of low parity was the most reported demographic barrier in the present study. This observation is logical because women want to achieve the desired number of children. Regarding the barriers related to the method itself, most of the participants complained of serious side effects because of contraceptive methods and consequently, the majority had the desire to have a more effective contraceptive method. Only 8% of the participants reported barriers related to the method itself in Kenya [31]. However, Aktun et al. reported a high discontinuation rate of contraceptive methods because of side effects [32]. Nearly three-quarters of the participants in this study said that written consent from the husband is mandatory by the health system to have contraceptive methods. This is in agreement with a study conducted in Pakistan which showed that the husband’s opinion is essential to have a family planning method [29] while other studies revealed that women have the authority regarding their reproductive decisions [18,33].

This study highlighted that, although the area of the study is considered a developing and relatively closed community, the rate of current and previous use of contraception is accepted. With exerting more efforts to increase population awareness, the contraceptive prevalence rate will equilibrate the national standard.

The present study had some limitations. First, the results of this study cannot be generalized because the participants were from the Aljouf region, not from all Saudi Arabia regions. Second, it is a cross-sectional study with more liability to interview and recall biases.

## 5. Conclusions

The present study showed that the rates of previous and current contraceptive use among the studied women were 32.2% and 45%, respectively. A significant association was shown between contraceptive use and women’s age, number of children, and monthly income. The most perceived barriers towards contraceptive utilization were cultural, demographic, medical, administrative, and barriers related to the method itself. More efforts are recommended to increase population awareness of the importance of family planning. Population awareness is very important because husbands have a vital role in directing their female partners to practice such family planning methods. Policymakers should work out barriers that hinder women from using different contraceptive methods. As the doctors were the primary source of information about contraception in this study, therefore the doctors should raise this topic more frequently with female patients who may benefit from considering this option.

## Figures and Tables

**Table 1:** Socio-demographic characteristics of women attending primary health centers in Aljouf region, Saudi Arabia.

Socio-Demographic Characteristics	No. (%) (n = 369)
**Age**	
≤25	42 (11.4)
26–30	56 (15.2)
31–35	138 (37.4)
>35	133 (36)
Mean ± SD (Range)	34.09 ± 6.42
**Residence**	
Rural	14 (3.8)
Urban	355 (96.2)
**Education**	
Illiterate	4 (1.1)
Primary/Preparatory	12 (3.3)
Secondary/Diploma	75 (20.3)
University/Postgraduate	278 (75.3)
**Occupation**	
Employed	194 (52.6)
Unemployed	175 (47.4)
**Children number**	
No	13 (3.5)
1–3	191 (51.8)
≥4	165 (44.7)
**Husband’s age**	
26–35	87 (23.5)
36–45	94 (25.5)
46–55	118 (32.0)
>55	70 (19.0)
**Years of marriage**	
<5 years	101 (27.4)
5–15 years	114 (30.9)
>15 years	154 (41.7)
**Monthly income**	
<5000 RS	58 (15.7)
≥5000 RS	311 (84.3)

**Table 2:** Correlates of contraceptive use among women attending primary health centers in Aljouf region, Saudi Arabia.

	User (n = 285) No. (%)	Non User (n = 84) No. (%)	COR (95% CI)	*p* Value	AOR (95% CI)	*p* Value
Age				0.000		0.000
≤25	25 (59.5%)	17 (40.5%)	1		1	
26–30	32 (57.1%)	24 (42.9%)	0.72 (0.40–1.37)		0.84 (0.26–2.67)	
31–35	108 (78.3%)	30 (21.7%)	0.51 (0.38–1.26)		1.67 (0.47–6.57)	
>35	120 (90.2%)	13 (9.8%)	6.06 (2.36–16.36)		4.52 (1.56–15.42)	
Residence				0.239		0.426
Rural	9 (64.3)	5 (35.7)	1		1	
Urban	276 (77.7)	79 (22.3)	1.03 (0.97–1.09)		0.71 (0.20–2.53)	
Education				0.476		0.189
Illiterate	2 (50)	2 (50)	1		1	
Primary/Preparatory	9 (75)	3 (25)	0.02 (0.01–0.05)		0.01 (0.02–0.06)	
Secondary/Diploma	61 (81.3)	14 (18.7)	0.13 (0.03–0.43)		0.27 (0.04–0.16)	
University/Postgraduate	213 (76.6)	65 (23.4)	0.03 (0.08–0.21)		0.04 (0.02–0.41)	
Occupation				0.835		0.089
Housewives	136 (77.7)	39 (22.3)	1		1	
Working	149 (76.8)	45 (23.2)	0.97 (0.77–1.23)		0.59 (0.33–1.04)	
Children number				0.000		0.003
No	5 (38.5)	8 (61.5)	1		1	
1–3	137 (71.7)	54 (28.3)	1.08 (0.61–2.35)		1.67(0.36–5.67)	
≥4	143 (86.7)	22 (13.3)	1.37 (1.75–2.18)		1.41(1.06–1.92)	
Husband’s age				0.849		0.212
26–35	64 (73.6%)	23 (26.4%)	1		1	
36–45	67 (71.3%)	27 (28.7%)	1.21 (0.42–2.04)		1.45 (0.23–7.98)	
46–55	100 (84.7%)	18 (15.3%)	0.53 (0.28–0.80)		0.74 (0.25–2.58)	
>55	54 (77.1%)	16 (22.9%)	0.68 (0.03–1.11)		1.38 (0.36–3.61)	
Years of marriage				0.014		0.182
<5 years	70 (69.3)	31 (30.7)	1		1	
5–15 years	85 (74.6)	29 (25.4)	2.03 (0.78–2.24)		3.81 (0.74–16.12)	
>15 years	130 (84.4)	24 (15.6)	1.45 (1.75–2.27)		1.87 (0.85–3.05)	
Monthly income				0.000		0.000
<5000 RS	33 (56.9)	25 (43.1)	1		1	
≥5000RS	252 (81)	59 (19)	1.25 (1.08–1.45)		2.29(1.24–8.27)	

**Table 3:** Barriers towards contraceptive utilization among women attending primary health centers in Aljouf region, Saudi Arabia.

Perceived Barriers	No. (%) (n = 203)
**Cultural barriers**	
Thinking that using contraceptive methods is bad behavior.	112 (55.2)
Childbearing is an easy process at a younger age.	162 (79.8)
Nontraditional contraceptive methods can endanger women’s health	199 (98)
Absence of female physicians.	82 (40.4)
The ideal number of children is not known.	106 (52.2)
**Demographic barriers**	
Desire to have more children because of low parity.	124 (61.1)
Contraceptive methods should be used by old women who do not want to be pregnant.	97 (47.8)
Having difficulty in becoming pregnant.	75 (36.9)
**Barriers related to the method itself**	
Desire to have a more efficient contraceptive method.	93 (45.8)
Failing in using different contraceptive methods.	57 (28.1)
Encountered serious side effects	96 (47.3)
**Psychosocial and physical barriers**	
Someone prevents you from using different contraceptive methods.	57 (28.1)
Always busy and involved in many tasks throughout the day.	83 (40.9)
The primary health center is away from my home.	34 (16.7)
**Medical barriers**	
Requesting the women to return more often than usual for examinations.	110 (54.2)
Informed written consent from the husband is mandatory to have contraceptive methods.	153 (75.4)
Hormonal contraceptive methods require women to be on her period.	131 (64.5)
**Administrative barriers**	
I did not attend any educational sessions about family planning	128 (63.1)
I did not hear or see any announcement about family planning.	58 (28.6)
Bad quality service.	78 (38.4)
Previous bad experience.	56 (27.6)
The negative attitude of the service provider.	32 (15.8)
Not considering privacy during the examination.	39 (19.2)

*Notes*: More than one barrier had been reported.
